# Plant-Based Diet for Glycemic Control, Insulin Sensitivity, and Lipid Profile in Type 2 Diabetes: A Systematic Review

**DOI:** 10.3390/foods14111919

**Published:** 2025-05-28

**Authors:** Siwatt Thaiudom, Kakanang Posridee, Sunthara Liangchawengwong, Chantira Chiaranai, Saranya Chularee, Aoitip Samanros, Anant Oonsivilai, Naruemol Singha-Dong, Ratchadaporn Oonsivilai

**Affiliations:** 1 Health and Wellness Research Unit, School of Food Technology, Institute of Agricultural Technology, Suranaree University of Technology, Nakhon Ratchasima 30000, Thailand; thaiudom@g.sut.ac.th (S.T.); posridee.ka@gmail.com (K.P.); 2 Institute of Nursing, Suranaree University of Technology, Nakhon Ratchasima 30000, Thailand; satharun@gmail.com (S.L.); chantira@sut.ac.th (C.C.); saranya.c@sut.ac.th (S.C.); 3 School of Nutrition and Dietitian, Institute of Public Health, Suranaree University of Technology, Nakhon Ratchasima 30000, Thailand; aoitip@sut.ac.th; 4 School of Electrical Engineering, Institute of Engineering, Suranaree University of Technology, Nakhon Ratchasima 30000, Thailand; anant@sut.ac.th

**Keywords:** type 2 diabetes mellitus (T2D), plant-based intervention, functional food, glycemic control, insulin sensitivity, lipid profile, systematic review

## Abstract

Background/Objective: Type 2 diabetes mellitus (T2D) is a chronic metabolic disorder characterized by hyperglycemia. Plant-based interventions have gained attention as potential complementary treatments alongside conventional therapies. This systematic review evaluates the efficacy of plant-based interventions in improving glycemic control, insulin sensitivity, lipid profiles, and other outcomes such as GLUT-4, Tumor Necrosis Facto-alpha, dietary inflammation index, plasma lipopolysaccharide, total antioxidant capacity, and malondialdehyde in individuals with T2D. Methods: We conducted a systematic search of PubMed, Scopus, and ScienceDirect databases to identify randomized controlled trials (RCTs) and observational studies. RCTs were used as an additional screening criterion. The review included studies on the effects of plant-based interventions, encompassing fruits, vegetables, herbs, spices, and their extracts. We analyzed data on glycemic control, insulin sensitivity, lipid profiles, and other metabolic markers. Results: Twenty-six studies were included in our analysis. Various interventions showed potential benefits, with improved glycemic control, insulin sensitivity, and lipid profiles. Specific interventions such as *Ziziphus jujuba* juice, black tea, caper fruit extract, and balanced diets were linked with positive outcomes. Based on the Functional Food Claim framework, all 26 studies met the quality criteria for novel foods. However, the novel food score varied, and results were inconsistent across different interventions. Conclusion: Although some plant-based interventions appear promising in managing T2D, the evidence remains inconclusive due to variability in study quality and methodology. Further high-quality RCTs are necessary to confirm these findings and to establish the optimal dosage, duration, and combinations of interventions for effective T2D management. Despite inconclusive results, few plant-based diets have promising outcomes. Healthcare providers, especially nurse case managers, can incorporate the findings of this study into their practice protocol to support self-management for individuals with TD2.

## 1. Introduction

Type 2 diabetes mellitus (T2D) is a chronic metabolic disorder characterized by persistent hyperglycemia, resulting from a combination of insulin resistance and impaired insulin secretion. This condition is a major global health issue, leading to increased morbidity and mortality through its contribution to serious complications such as cardiovascular disease, kidney dysfunction, neuropathy, retinopathy, and diabetic foot problems. According to a recent report by the International Diabetes Federation (IDF), approximately 537 million adults worldwide were living with diabetes in 2021. This number is expected to rise to 783 million by 2045, underscoring the urgent need for effective management strategies. T2D disproportionately impacts low- and middle-income countries (LMICs), where limitations in healthcare accessibility further exacerbate the challenges faced by these populations [1,2,3].

Traditional management of T2D involves lifestyle interventions, such as dietary modifications, increased physical activity, and weight management, alongside pharmacological treatments, including metformin, sulfonylureas, and glucagon-like peptide-1 (GLP-1) receptor agonists [4]. Although these methods are established, there are ongoing challenges in maintaining long-term glycemic control and minimizing complications. Additionally, pharmacological options may pose side effects or remain out of reach for patients in resource-limited settings [1,2,3].

Self-management is widely recognized as a crucial aspect of improving self-management behaviors and clinical outcomes, particularly for individuals with non-communicable diseases (NCDs) such as diabetes and hypertension [5,6]. For individuals with T2D, self-management involves actively participating in self-care through lifestyle changes, including maintaining a healthy diet, engaging in regular exercise, monitoring blood glucose levels, adhering to medication regimens, and resolving diabetes-related issues [7,8]. Healthy food is essential for managing blood sugar levels and preventing complications, yet it remains a challenge for many. A case management approach provides support and guidance to individuals with T2D, empowering them to make better decisions and enhance their self-management skills [7]. Shin and colleagues reported that the case management program motivated patients to improve their knowledge, self-management skills, health behaviors, and healthcare utilization [9]. Patients’ self-care behaviors were found to have a positive association with provider recommendations, which were directly influenced by diabetes distress, health literacy, family history of diabetes, and prior diabetes education [7,8,9].

The Foods with Function Claims (FFC) system, introduced in Japan in April 2015, revolutionized the health food market by offering a more accessible pathway for manufacturers to make health claims. Unlike the stricter pre-existing systems (Foods for Specified Health Uses; FOSHU, and Foods with Nutrient Function Claims; FNFC), which involved lengthy and costly approval processes, the FFC allows companies, especially SMEs, to market health-benefiting products by simply notifying the Consumer Affairs Agency (CAA). Key to the FFC system is the manufacturer’s responsibility to possess and submit scientific evidence—derived from clinical trials or systematic reviews—to substantiate the product’s claimed health effects and safety. The CAA publicly disseminates this information and conducts post-market surveillance. This deregulation significantly boosted the health food sector, leading to over 3500 FFC-labeled products by 2020. Furthermore, the system encourages research into agricultural products with inherent health benefits, such as β-cryptoxanthin-rich Satsuma mandarins for bone health and O-methylated catechin-rich Bennifuki tea for allergy reduction, thereby enhancing the economic value and quality of local agricultural and food products from farm to consumer. Ultimately, the FFC serves as a crucial tool for promoting innovation and value-addition in Japan’s agricultural and food industries [10].

Amid these challenges, functional foods have emerged as promising adjuncts in the management of T2D. Defined as foods that provide health benefits beyond basic nutrition, functional foods, particularly those from plant-based sources, have attracted interest for their potential to enhance glycemic control, insulin sensitivity, and lipid profiles. Such foods include fruits, vegetables, herbs, spices, and plant extracts rich in bioactive compounds like polyphenols, flavonoids, and dietary fibers. These compounds are known to modulate glucose metabolism, boost insulin sensitivity, and reduce inflammation—crucial factors in the pathophysiology of T2D [3].

However, the existing research on plant-based diets has significant limitations. Studies frequently differ in intervention type, dosage, duration, and participant characteristics, leading to inconsistent results and limited generalizability. Additionally, many studies do not meet high methodological standards, often lacking proper randomization, control groups, or comprehensive outcome measures, which makes drawing definitive conclusions challenging [11]. These issues underscore the need for systematic evaluations to ascertain the efficacy and practical applicability of plant-based interventions in managing T2D.

This systematic review addresses these limitations by synthesizing evidence from a broad spectrum of high-quality studies on plant-based dietary interventions for individuals with T2D. It specifically examines the effects of these interventions on glycemic control and insulin sensitivity as primary outcomes, and lipid profiles as secondary outcomes, providing a thorough analysis of their potential role in disease management. The healthcare Functional Food Claim Japan (FFC) rating system was employed to evaluate the quality of the studies included, ensuring a rigorous assessment of the evidence supporting novel food claims for industry applications [10,12]. The review provides a comprehensive analysis of the potential role of these interventions in managing the disease as well as regulatory landscape of novel food products, with a particular focus on the FFC Japan, where the regulatory framework for functional food claims is well-established and serves as a model for other countries [12].

- **Aims**

The primary objectives of this study were to identify plant-based diets that effectively reduce blood glucose levels in individuals with type 2 diabetes. Additionally, the secondary objective was to assess the quality of food claims based on the FFC of the Japanese Consumer Affairs Agency (CAA) [13].

- **Research questions**

Under the FFC standard, can a plant-based diet effectively reduce glycemic levels among individuals with T2D?

## 2. Materials and Methods

This review is guided by the Preferred Reporting Items for Systematic Reviews and Meta-Analyses (PRISMA) guidelines to identify, select, appraise, and synthesize studies [14]. In addition, the review included a risk of bias assessment using the Cochrane Risk of Bias 2 (ROB 2) tool as well as the Japanese FFC rating system [10,11,12,13,14,15]. Details are given below.

### 2.1. Search Strategy

To identify relevant studies, we conducted a systematic literature review using the PubMed, Scopus, and ScienceDirect databases. The search focused on articles published between 1 January 2010, and 23 December 2023, to emphasize current research. Search terms were developed using the PICO framework (Population, Intervention, Comparison, Outcome) to ensure precision and relevance. Keywords such as “diabetes mellitus Type II”, “plants”, “extract”, and “clinical trial” were combined using Boolean operators (AND, OR) to create search strings like “(diabetes mellitus Type II OR T2DM) AND (plants OR plant-based interventions) AND (RCT OR clinical trial)”. The initial search was conducted on 23 July 2023, with updates on 15 November 2023 and 23 December 2023. Full search terms are detailed in Supplementary Materials.

Following the database searches, the Rayyan platform was used to manage and screen the results systematically. This tool helped in removing duplicates, organizing citations, and facilitating collaboration among reviewers during the study selection process.

Protocol Registration

This systematic review did not register under PRISMA but registered with Rayyan (Registration number: 844955).

### 2.2. Selection Criteria

This review included studies that met the following criteria: participants were adults (18 years or older) diagnosed with T2D. Interventions had to involve plant-based elements, such as whole plants, extracts, herbs, or functional foods. Studies were required to report outcomes related to glycemic management (e.g., glycated hemoglobin or hemoglobin A1C (HbA1c), fasting blood glucose), insulin sensitivity, or lipid profiles. Eligible study designs included randomized controlled trials (RCTs), non-RCTs, and observational studies with a comparator group. RCTs were used as an additional screening criterion. Only English-language publications providing sufficient data were included. Studies focusing on type 1 diabetes, gestational diabetes, or conditions co-occurring with T2D were excluded, as were studies without a comparison group, those lacking adequate data for analysis, or those evaluating non-plant-based diet.

### 2.3. Data Extraction

All titles from the database searches were initially screened by the primary researchers (ST, RO, CC, and NS) to discard irrelevant studies. Nine researchers (ST, KP, SL, CC, SC, AS, AO, NS, and RO) independently screened the titles and abstracts of potentially eligible studies using a predefined eligibility checklist to identify those suitable for full-text review. The full texts of 91 selected studies were then retrieved and reviewed by three reviewers (ST, KP, and RO) to determine final inclusion. Data extraction from these studies was performed by the primary researchers, with verification by secondary and tertiary researcher groups. To ensure data accuracy, any discrepancies were collaboratively discussed by all nine reviewers until a unanimous agreement was reached.

### 2.4. Quality Assessment

#### 2.4.1. Risk of Bias Assessment

The ROB 2 tool will be used to assess the risk of bias in RCTs [16]. Domains include (1) bias arising from the randomization process, (2) bias due to deviations from intended interventions, (3) bias due to missing outcome data, (4) bias in measurement of the outcome, and (5) bias in selection of the reported result. Three reviewers independently assessed the risk of bias. Disagreements were resolved through discussion.

#### 2.4.2. Functional Food Claim Japan Quality Assessment

Japan has a unique regulatory framework for functional foods. This system, launched in 2015 by the CAA, allows manufacturers to make health claims on food labels without needing pre-approval, provided they submit scientific evidence and notify the CAA. The FFC system requires that (1) companies must submit a dossier with scientific evidence supporting the claimed functionality; (2) the claims must be based on well-designed clinical trials or systematic reviews; and (3) transparency is maintained through public access to submitted data on the CAA website [10]. The system ensures that functional claims are evidence-based [12].

The quality of included studies was assessed based on five components: (1) the materials used, their preparation, and any relevant analytical methods; (2) study population; (3) study design, which refers to the overall structure of the study, such as an RCT or a case-control study; (4) research results and conclusions; and (5) health claims validation. The reliability of studies was ranked on a three-level scale (−2 to 0) for each component. Furthermore, the overall levels for each component were used to define the strength of the study, classified as A–E; A: clear and well-founded (all five components at level 0), B: positively grounded (all five components at level −1), C: suggestively grounded (all five components at level −2), D: insufficient evidence, and E: negative evidence [14,15]. Data curation followed the categorization by Mohamed et al., 2019 [4], alongside the FFC Bulletin. Criterion 3 of the FFC, which requires consensus among three food technology background authors for record selection, was applied to minimize selection bias [11,13].

Moreover, for nutrient function claims and other function claims submitted to the Thai FDA, robust evidence, primarily from human clinical trials, especially those focusing on specific population groups, is considered the most compelling. This may be supported by evidence from in vitro studies and epidemiological research [12].

## 3. Results

**Figure 1:**
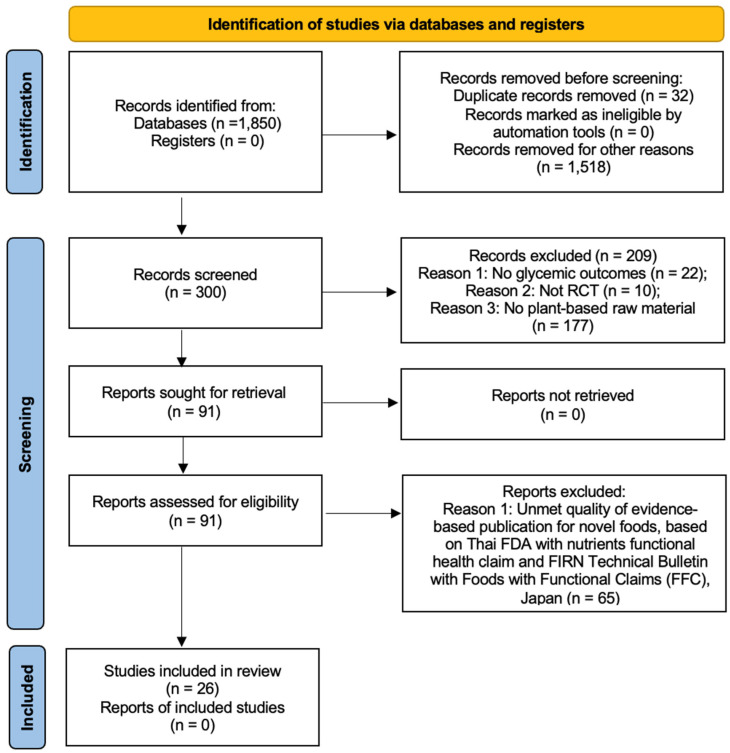
PRISMA diagram [15].

The search strategy identified 1850 unique publications. After initial screening, 300 abstracts were assessed for relevance. Ninety-one of these abstracts warranted full-text reviews. Ultimately, 30 studies met our inclusion criteria and were selected for detailed analysis (Figure 1). The interventions in these studies varied in terms of the number and duration of sessions; however, their primary focus was on the effects of plant or plant extract administration on blood chemistry, particularly HbA1c levels, in individuals with T2D.

This systematic review adhered to the methodological standards outlined in the PRISMA guidelines. Initially, 1850 non-duplicated records were screened. Following the assessment of 300 abstracts, 91 publications underwent full-text evaluation. Of these, 26 studies fulfilled the inclusion criteria for this review (Figure 1). Despite variation in the number and duration of treatment sessions among the studies, all interventions predominantly examined the impact of plants or plant extracts on blood chemistry, specifically targeting HbA1c levels in individuals with T2D.

### 3.1. Study Characteristics

Twenty-six studies were analyzed in this systematic review. All 26 studies [7,8,9,10,11,12,13,14,15,16,17,18,19,20,21,22,23,24,25,26,27,28,29,30,31,32] were RCTs. The duration of interventions ranged from 8 weeks to 2 years. A significant portion of this research has been conducted in diverse regions, with fifteen studies originating from Iran, significantly contributing to the existing knowledge base [16,17,19,21,22,23,26,27,28,29,30,32,33,34,35]. Additionally, research efforts have extended to the UK [36,37], Germany [38,39], and Canada [18,40], with each country contributing two studies. Furthermore, single studies have been conducted in China [20], USA [24], Ireland [25], Kuwait [31], and Italy [41]. Most participants were aged between 30 and 69 years (*n* = 21, 81%), while those aged 70 years and above constituted 19% (*n* = 5) of the study population [17,18,19,20,21,22,23,24,25,26,27,28,29,30,31,32,33,34,36,37,38,39,40,41].

In this review, the majority of studies (53%) used HbA1c as the primary outcome measure, with observed effect sizes (Cohen’s d) ranging from 0.1 to 1.0. Blood lipid profiles were commonly assessed as a secondary outcome.

### 3.2. Quality Assessment

Initially, 91 studies were evaluated for quality using the Functional Food Claim Japan (FFC) rating system [10,11]. The quality of the included studies was assessed based on the five components discussed previously. Applying FFC’s third criterion minimized selection bias, particularly ensuring consensus among three food technology background authors for record selection. Ultimately, twenty-six studies were selected for inclusion, with nine achieving a ‘Grade B’ [16,17,18,19,20,21,22,23] and seventeen classified as ‘Grade C’ [24,25,26,27,28,29,30,31,32,33,34,36,37,38,39,40,41].

### 3.3. Impact of the Plant or Plant Extract Intervention on Blood Sugar and HbA1c

Table 1 and Table 2 summarizes the findings from twelve studies graded as “Grade B” that explored the effects of functional food interventions on individuals with T2D. These studies encompassed various interventions, including pomegranate seed oil [16], saffron [17], sesame or canola oil [18], nano-curcumin [19], and a diet incorporating 100 g/d of germinated brown rice (GBR) [20]. The duration of these interventions varied. Pomegranate seed oil supplementation [16] significantly improved fasting blood sugar and insulin sensitivity. Nano-curcumin supplementation [19] reduced blood sugar, HbA1c, and neuropathy symptoms. The GBR intervention [20] decreased inflammation, blood sugar, and lipid levels while increasing beneficial fatty acids. In the control group, placebo rusk powder improved blood sugar control through SIRT1 activation. Additionally, oligofructose-enriched inulin reduced blood sugar, HbA1c, and inflammation markers.

The studies included a diverse group of participants, primarily adults diagnosed with T2D. The interventions tested were pomegranate seed oil [16], saffron [17], sesame or canola oil [18], nano-curcumin [19], and a diet incorporating 100 g/day of GBR [20]. These interventions varied in duration from one week to three months. Pomegranate seed oil supplementation [16] significantly improved key outcomes such as fasting blood sugar and insulin sensitivity. Saffron supplementation [17] effectively lowered blood sugar levels and reduced inflammation markers. Additionally, supplementation with sesame or canola oil [10] enhanced insulin sensitivity and improved lipid profiles.

The study conducted by Sauder in the United States in 2015 [24] was a randomized, crossover, controlled trial involving 30 adults with T2D. This four-week study compared the effects of a nutritionally balanced diet, including pistachios, to a similar diet, excluding pistachios. Although no significant differences were observed in primary outcomes such as fasting blood sugar, insulin levels, and inflammatory markers, the study did find that fructosamine levels were notably lower in the pistachio group. Additionally, the inclusion of pistachios resulted in significant reductions in secondary outcomes like total cholesterol, HDL cholesterol, and triglycerides compared to the diet without pistachios [24].

The 2021 randomized, double-blind, placebo-controlled crossover study involved 131 adults with T2D and investigated the impact of inulin-type fructan (ITF) prebiotic supplementation on glycemic control and incretin hormone levels. Participants were randomly assigned to receive 16 g/day of ITF or a placebo for six weeks. The results showed a significant decrease in glycated hemoglobin (HbA1c) in the ITF group compared to the placebo group (−0.56% vs. −0.07%, 95% CI: −0.25 to −0.86% vs. −0.15 to 0.29%), which was the primary endpoint. However, ITF supplementation did not significantly affect GLP-1, glucagon-like peptide-2 (GLP-2), fasting glucose, or insulin levels [22].

**Figure 2:**
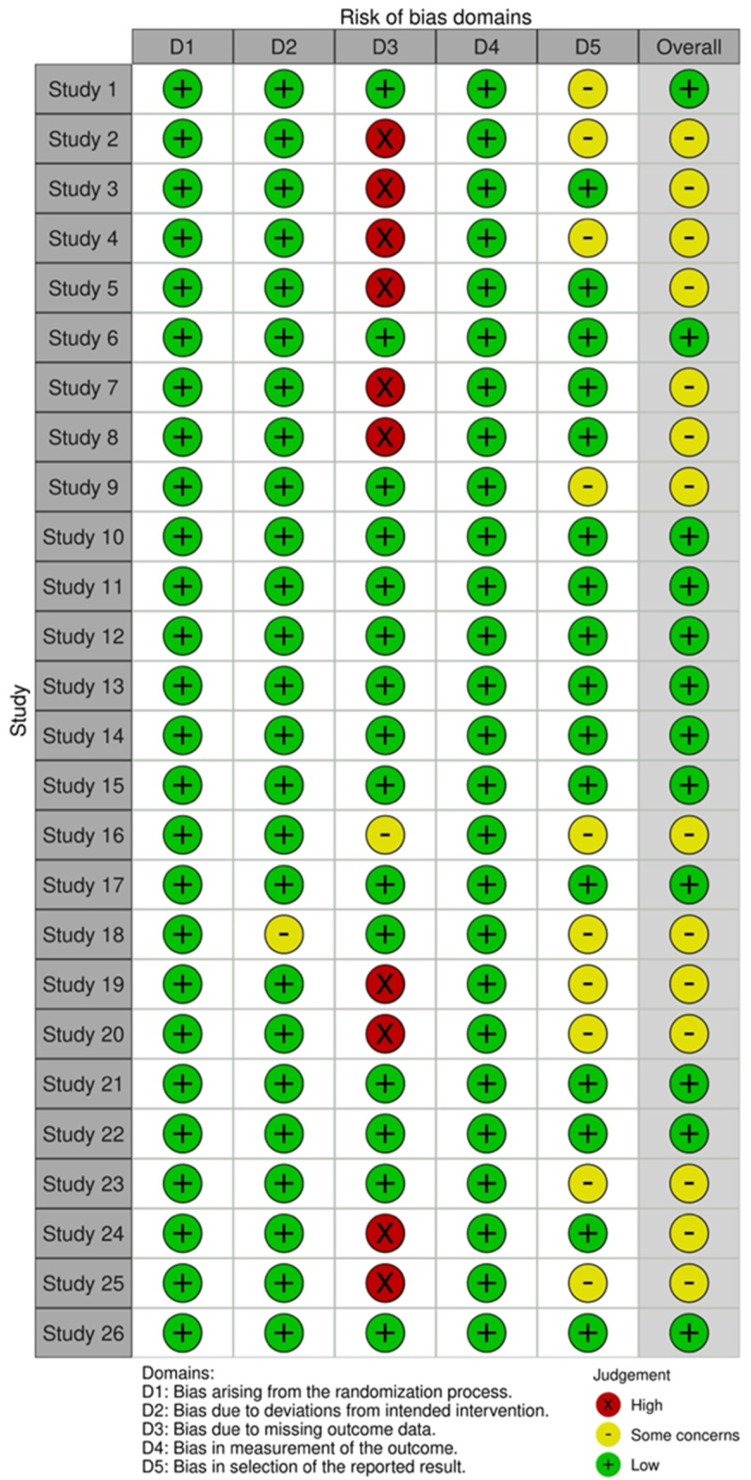
Risk of bias assessment results using the Cochrane risk of bias (RoB 2) tool for randomized clinical trial studies [42].

**Figure 3:**
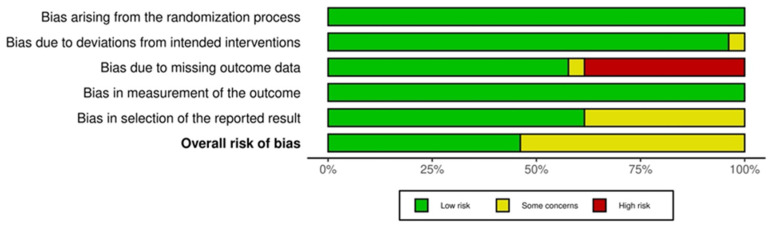
Risk of bias summary graph for randomized controlled trials [42].

Figure 2 and Figure 3 show the quality assessment (Revised tool for Risk of Bias in randomized trials) for the included RCTs, generated by robvis [42]. RCT domain 1 (D1), Bias arising from the randomization process; D2, Bias due to deviation from the intended intervention; D3, Bias due to missing outcome data; D4, Risk of bias in measurement of the outcome; D5, Risk of bias in selection of the reported result [15]. Domains 1 and 3 show no risk of bias; the majority of domain 2 have some concern. Despite the risk of bias raised due to missing outcome data, none of the included studies were categorized as “high risk”.

Functional food interventions such as beetroot juice supplementation [17], fenugreek seeds [18], pomegranate seed powder [19], okra powder [20], saffron supplementation [21], and chicory inulin [41] may improve glycemic control, insulin sensitivity, lipid profiles, and other metabolic parameters in individuals with T2D. The findings from these studies demonstrate various benefits, including improved glycemic control and reduced insulin resistance with ZJF juice [22], enhanced fasting blood glucose levels and HbA1c with black tea [23], and decreased fasting blood glucose alongside improved lipid profiles with caper fruit extract [24]. Additionally, increased regulatory T cells and reduced pro-inflammatory cytokines were noted with black tea consumption [23].

The analysis of the provided studies indicates that functional food interventions, such as flaxseed oil [26], soy nuts [27], aged garlic extract [28], and flavanol-rich cocoa powder [29], may positively affect lipid profiles, glycemic control, insulin sensitivity, blood pressure, endothelial function, blood flow, vascular function, muscle mass, and strength in individuals with T2D. However, gelatin capsules and aged garlic extract did not demonstrate significant benefits in the reviewed studies. Furthermore, the effects of flavanol-rich cocoa powder on T2D remain inconclusive.

The analysis of the provided studies suggests that functional food interventions—including resveratrol [30], modified oat [31], insoluble fiber, and oat-derived β-glucan—may improve glycemic control, insulin sensitivity, lipid profiles, and other metabolic parameters in individuals with T2D. Significant findings from these studies include enhanced glycemic control and insulin sensitivity with D-allulose; reduced blood glucose levels with resveratrol; improved control over glycemic levels and increased satiety with whey protein; better glycemic management and insulin responsiveness with modified oat; decreased glucose levels when using white rice; lowered HbA1c, fasting plasma glucose, and 2-h plasma glucose with specific carbohydrate and fat combinations; improved postprandial peak glucose levels with D-allulose; consistent reduction of blood glucose levels with resveratrol; diminished daily hyperglycemia and enhanced overall glycemic control with whey protein preloads; lowered total cholesterol with oat-derived β-glucan; and significant reductions in HbA1c levels, along with improved insulin sensitivity and hepatic insulin clearance with insoluble fiber.

## 4. Discussion

The provided research suggests that a variety of natural foods and dietary components hold promise for improving metabolic health in individuals with T2D. By targeting key mechanisms involved in the disease process, these foods may contribute to improved glycemic control, lipid profiles, and overall cardiovascular health. However, these findings should be interpreted with caution. There is a need for more rigorous research and a comprehensive approach to managing T2D. Individuals with T2D should consult their healthcare providers before making significant dietary changes.

The review offers an extensive overview of functional foods potentially beneficial for T2D management, yet the evidence is limited by significant flaws. The studies demonstrate considerable heterogeneity in design, population, dosages, and durations, which complicates comparisons and generalizations. Broad claims regarding improvements in glycemic control and inflammation are often nonspecific, with insufficient discussion of the underlying mechanisms or clinically significant outcomes. The lack of consistently reported control groups and the potential for publication and funding biases further undermine the strength of the evidence. Additionally, the emphasis on biomarker changes without corresponding long-term outcome data, along with an absence of standardized recommendations and consideration of individual variability, underscores the necessity for more rigorous research. This includes conducting large-scale RCTs to verify the efficacy and determine the optimal use of these functional foods.

The search strategy employed in this review exhibits several limitations that may affect its comprehensiveness. Primarily, the reliance solely on PubMed, Scopus, and ScienceDirect potentially excludes relevant studies available in other valuable databases such as Embase and the Cochrane Library, thus limiting the scope of the review. Additionally, restricting the search to English-language publications introduces a language bias, possibly overlooking significant research published in other languages. Although the PICO framework was used, the broad search terms like “plants” lacked the necessary specificity to retrieve targeted research effectively. A more detailed approach, using specific plant names or phytochemicals, would likely have yielded more precise results. Moreover, while updates were conducted, the intervals between these updates might have missed recent studies, especially in the rapidly evolving field of nutrition research.

The selection criteria employed in this review also raise several concerns regarding the validity and generalizability of its findings. Including a range of study designs from RCTs to observational studies introduces significant heterogeneity. Observational studies, in particular, are prone to confounding variables and generally offer weaker causal inferences. Excluding studies involving co-occurring conditions, while practical, limits the applicability of the review’s conclusions to real-world scenarios where comorbidities are common. The subjective criterion of “sufficient data” for inclusion lacks clarity and consistency, which could lead to biased study selection. A more objective and predefined definition of what constitutes sufficient data would have been preferable. Furthermore, the vague reference to the use of RCTs as “additional screening criteria” requires clarification. It remains unclear whether RCTs were used to refine initial screenings or to prioritize certain study designs, a distinction that could significantly influence the conclusions drawn from the review.

The data extraction and quality assessment processes within this review reveal several weaknesses that raise concerns about the reliability of the synthesized evidence. The use of the Functional Food Claim Japan (FFC) rating system provides a framework but may not be universally suitable for all nutrition research and is susceptible to subjective biases. The failure of the review to explicitly report inter-rater reliability scores, such as kappa statistics, for data extraction and quality assessment undermines its credibility. Moreover, while the FFC system assesses study quality, the absence of detailed critical appraisals for each study limits the evaluation of bias risk within individual studies.

The findings of this review, which highlight the potential benefits of various functional foods for T2D, necessitate a cautious approach in clinical practice, policy formulation, and future research. Clinically, these functional foods should be considered complementary therapies, requiring personalized recommendations and careful monitoring, complemented by robust patient education. From a policy perspective, the development of evidence-based dietary guidelines and regulated food labeling should be considered, albeit with a strong emphasis on securing further research funding. Future research should prioritize rigorous, large-scale RCTs to confirm efficacy, investigate mechanisms of action, assess long-term outcomes, determine optimal dosages, and explore effects specific to different populations. It is also essential to standardize quality assessment tools. Ultimately, translating these promising findings into tangible clinical and public health benefits depends on robust, well-designed research.

This review on functional foods for type 2 diabetes (T2D) management is significantly limited by the heterogeneity and methodological flaws in the underlying evidence. Key limitations include considerable variability in study designs (from RCTs to observational), diverse populations, dosages, and durations, which hinder direct comparisons and generalizability. Furthermore, the outcomes reported often lack specificity and long-term clinical relevance, focusing on biomarker changes without corresponding patient outcomes. The search strategy itself is limited by relying on a few databases and English-only publications, potentially omitting significant research. Concerns also arise from the broad and subjective selection criteria, including studies prone to confounding and unclear definitions of “sufficient data”. Finally, weaknesses in data extraction and quality assessment, such as the absence of inter-rater reliability, further compromise the synthesized evidence. These collective limitations underscore the urgent need for rigorous, large-scale RCTs to robustly confirm efficacy, investigate mechanisms, and determine optimal use across diverse T2D populations.

Under the FFC framework, several studies have presented practical strategies for using plant-based diets in T2D clinical management, which have been found to be highly acceptable in medical contexts [43]. Clinicians may consider suggesting that their T2D patients adopt a plant-based diet, such as nano-curcumin, green cardamom, and pistachio. The likelihood of certain nano-curcumin formulations effectively treating T2D is cautiously optimistic, with significant potential. Traditional curcumin faces challenges in achieving good bioavailability, which nanotechnology aims to address. Clinical trials on nano-curcumin have yielded promising results, demonstrating significant reductions in fasting blood glucose (FBG), HbA1c, and inflammatory markers (such as CRP). These trials have also shown improvements in lipid profiles and suggest potential benefits for managing diabetic complications like neuropathy. However, more extensive, long-term trials are necessary to standardize the formulations, confirm optimal dosing, and establish its role as a complementary therapy in conjunction with conventional treatments. Ongoing self-management support, education, and follow-up can help patients achieve and maintain dietary changes for glycemic control.

## 5. Conclusions

This systematic review aimed to evaluate the efficacy of plant-based interventions in managing T2D. A total of 30 studies, primarily RCTs, were included in the analysis. These studies covered a wide range of plant-based products, including fruits, vegetables, herbs, spices, and their extracts.

The findings suggest that certain plant-based interventions may beneficially impact glycemic control, insulin sensitivity, lipid profile, and other metabolic parameters in individuals with T2D. However, the evidence remains inconsistent, and the quality of the studies varies. Some interventions, such as pomegranate seed oil, nano-curcumin, and GBR, showed promising results in improving blood sugar control and insulin sensitivity. Additionally, other interventions, including saffron, sesame or canola oil, and pistachios, also demonstrated potential benefits for glycemic control and lipid profiles.

## Figures and Tables

**Table 1:** Overview of the eligible studies examining the effects of functional foods interventions for people with type 2 diabetes mellitus (T2D) categorized as Grade B.

Reference No.	Participant Enrollment and Retention	Clinical Subgroup	Average Age (Standard Deviation or Range), in Years	Intervention Details	Intervention Protocol	Intervention Details for the Control Group	Treatment Protocols for the Control Group and the Therapist	Effect Size of HbA1c (d)	Secondary Outcomes (Intervention vs. Control)	Time After Initial Assessment (Weeks)	Quality (Novel Food Score)
16	60/52	60 Obese T2D	30–50	Clinical/ Pomegranate seed oil supplementation (PSO)	Consumed 3 capsule/day (1 g PSO)	No intervention	No intervention	No significant changes of HbA1c	-PSO group demonstrated significant improvements in both fasting blood sugar (FBS) and the quantitative insulin sensitivity check index. -PSO treatment led to increased GLUT-4 gene expression in diabetic patients without any observed adverse effects.	8	B
17	60/60	60 T2D	50–60	Clinical/ Saffron supplement-ation	Consumed 1 capsule/day (100 mg saffron powder)	No intervention	Starch capsule 100 mg/day	N/A	-Fasting Blood Glucose (FBG): After 8 weeks, saffron group exhibited a statistically significant reduction in FBG levels compared to the placebo group (130.93 ± 21.21 vs. 135.13 ± 23.03 mg/dl, *p* = 0.012). -Tumor Necrosis Factor-alpha (TNF-α): Saffron supplementation led to a significant decrease in serum TNF-α levels compared to placebo (114.40 ± 24.28 vs. 140.90 ± 25.49 pg/mL, *p* < 0.001). -Gene Expression: The mRNA levels revealed that saffron significantly downregulated the expression of both TNF-α (*p* = 0.035) and IL-6 (*p* = 0.014).	8	B
18	51 males, 51 females/ 46 males, 49 females	95 T2D	18–60	Clinical/Sesame (SO), canola (CO), and sesame–canola (SCO: a blend) oils	SO, CO, or SCO (40% SO and 60% CO) substituted common edible oils/9 weeks with 4 weeks wash out period	No intervention	No intervention	N/A	-All treatment oils resulted in significant improvements in fasting serum insulin (FSI) and insulin sensitivity (HOMA2-%S) (*p* < 0.05). -SO and SCO led to favorable changes in insulin resistance (HOMA2-IR) and quantitative insulin sensitivity check index (QUICKI) (*p* < 0.05). -CO and SCO treatments revealed significant decrease in beta-cell function (HOMA2-%B) (*p* < 0.05). -FSI and HOMA2-IR were decreased after SO compared to CO in males (*p* = 0.024). -Serum gamma-glutamyltransferase (GGT) was significantly lower following SO compared to CO in females (*p* = 0.02), also no significant difference (*p* = 0.058).	9	B
19	80/80	80 T2D	30–60	Clinical/nano-curcumin	Nano-curcumin80 mg/day	placebo capsules	Placebo capsules	Significant reduction in HbA1c (*p <* 0.001)	-Nano-curcumin significantly lowered FBS levels (*p* = 0.004). -Nano-curcumin significantly improved neuropathic symptoms compared to placebo group by lower total neuropathy scores (*p <* 0.001), total reflex scores (*p* = 0.04), and temperature (*p =* 0.01).	8	B
20	112/61 females, 51 males	112 T2D	18–70	Clinical/Germinated brown rice (GBR)	Diet with 100 g GBR/day for 3 months (*n* = 56)	No Intervention (*n* = 56)	No Intervention (*n* = 56)	N/A	-GBR group reduced in mean dietary inflammation index (DII), a positive impact on mitigating inflammation. -GBR significantly lower FBG, HbA1c, total cholesterol (TC), and high-density lipoprotein (HDL). -GBR intervention led to alterations in fatty acid composition, with significant increases observed in n-3 polyunsaturated fatty acids (PUFAs) and the n-3/n-6 PUFA ratio. -GBR group elevated levels of n-3 metabolites, including RVE, MaR1, and PD1, which are associated with reduced inflammation.	12	B
21	83/83	83 Obese T2D	30–60	Clinical/green cardamom	Received 3 g of green cardamom/ 10 weeks	Clinical	Received 3 g rusk powder/ 10 weeks	HbA1C Decreased (−0.4%)	-A significant decrease in serum HbA1c (−0.4%), insulin (−2.8 μIU dL^−1^), HOMA-IR (−1.7), and TG (−39.9 mg dL^−1^), and an increase in SIRT1 (2.3 ngmL^−1^) was observed in cardamom group. -No significant changes (*p <* 0.05) in serum TC, HDL-c, and low density lipoprotein (LDL-c) levels.	10	B
22	70/54	54 (M = BMI > 25 kg/m^2^ but <35 kg/m^2^ with T2D)	20–65	Clinical/oligofructose-enriched inulin	Received 10 g oligofructose-enriched inulin/day (Fruta fit IQ, Sensus, Borchwef 3, 4704 RG Roosendaal, Netherlands/8 weeks	Clinical	Received 10 g maltodextrin/day as a placebo (Jiujiang Hurirong Trade Co., Ltd, JiuJiang, China)	N/A	-Oligofructose-enriched inulin supplementation led to a significant reduction in FBS levels (19.2 mg/dL; 9.50%), HbA1c (1.0%; 8.40%), interleukin-6 (IL-6) (1.3 pg/mL; 8.15%), TNF-α (3.0 pg/mL; 19.80%), and plasma lipopolysaccharide (LPS) (6.0 EU/mL; 21.95%) compared to maltodextrin (*p <* 0.05). -Decreases were observed in interferon-gamma (IFN-γ) (0.3 pg/mL; 16.50%) and high-sensitivity C-reactive protein (hs-CRP) (3.9 ng/mL; 31.70%), while interleukin-10 (IL-10) levels increased (0.4 pg/mL, 11.50%).	8	B
23	56/56	56 T2D and CHD	35–70	Clinical/resveratrol	-Received 500 mg resveratrol /day for 4 weeks	No intervention	No intervention	N/A	-Resveratrol reduced fasting glucose (*β* −10.04 mg dL^−1^; 95% CI, −18.23, −1.86; *p* = 0.01), insulin (*β* −1.09 μIU mL^−1^; 95% CI, −1.93, −0.24; *p* = 0.01), and insulin resistance (*β* −0.48; 95% CI, −0.76, −0.21; *p* = 0.001). -Significantly increased insulin sensitivity (*β* 0.006; 95% CI, 0.001, 0.01; *p* = 0.02). Resveratrol significantly increased HDL cholesterol levels (*β* 3.38 mg dL^−1^; 95% CI, 1.72, 5.05; *p* < 0.001) and significantly decreased the total-/HDL cholesterol ratio (*β* −0.36; 95% CI, −0.59, −0.13; *p* = 0.002). -Resveratrol caused a significant increase in total antioxidant capacity (TAC) (*β* 58.88 mmol L^−1^; 95% CI, 17.33, 100.44; *p* = 0.006) and a significant reduction in malondialdehyde (MDA) levels (*β* −0.21 μmol L^−1^; 95% CI, −0.41, −0.005; *p* = 0.04). -Resveratrol upregulated PPAR-γ (*p* = 0.01) and sirtuin 1 (SIRT1) (*p =* 0.01) in the peripheral blood mononuclear cells (PBMCs).	4	B
24	30/30	30 T2D post menopause women BMI of 18.5–45.0 kg/m^2^	40–74	Clinical/pistachios	Consumed nutritionally adequate diets with (contributing 20% of total energy) pistachios for 4 weeks each, separated by a 2-week washout. (Fresno, CA, USA)	No intervention	Normal diet	Decreased HbA1c by 0.4%	-Pistachio improved fasting glucose levels. -Significant reductions in fasting glucose, insulin, and HOMA-IR with pistachio intake.	2	B

Legend for acronyms. BMI = Body Mass Index; HbA1c = glycated hemoglobin, the average level of blood glucose over the past 2–3 months.

**Table 2:** Overview of the eligible studies examining the effects of functional foods interventions for people with type 2 diabetes mellitus (T2D) categorized as Grade C.

Reference No.	Participant Enrollment and Retention	Clinical Subgroup	Average Age (Standard Deviation or Range), in Years	Intervention Details	Intervention Protocol	Intervention Details for the Control Group	Treatment Protocols for the Control Group and the Therapist	Effect Size of HbA1c (d)	Secondary Outcomes (Intervention vs. Control)	Time After Initial Assessment (Weeks)	Quality (Novel Food Score)
25	46/38	38 T2D	54	Clinical/concentrated beetroot juice (BJ)	Consumed BJ 24 mL/day/12 weeks	No intervention	No intervention	No significant between two groups (*p =* 0.05)	-Plasma nitric oxide (NO) had a higher non -significant increase (8.57 ± 23.93 vs. 2.31 ± 15.98, *p* = 0.128). -Significant reductions in plasma insulin (14.55 ± 7.85 vs. 10.62 ± 6.96, *p =* 0.014) and HOMA-B (3.96 ± 0.83 vs. 3.63 ± 0.75, *p* = 0.038). -Significant reduction in HDL-C (70.81 ± 11.24 vs. 65.44 ± 6.46, *p =* 0.058) were observed in the control group after 12 weeks. -Diastolic blood pressure (DBP) was significantly reduced in the BJ group compared with the baseline (74.73 ± 16.78 vs. 72.36 ± 16.09, *p* = 0.046). -No significant effect on the levels of fasting plasma glucose (FPG), HbA1c, HOMA-β, HOMA-IR, TC, LDL, HDL, triglycerides (TG), and blood pressure (BP) was observed.	NA	C
26	90/88	88T2D	40–41	Clinical/powdered whole fenugreek seeds	Consumed powdered whole fenugreek seeds 10 g/day /8 weeks	Placebo	Consumed wheat starch 5 g/day	Significant decreased (*p =* 0.0001)	-Significant decrease was observed in FPG levels (*p =* 0.007), serum insulin concentrations (*p* = 0.03), TC levels (*p =* 0.005), and TG levels (*p =* 0.0001).	NA	C
27	65/60	60T2D	62–63	Clinical/Pomegranate seed powder (PSP)	Consumed PSP 5 g twice daily/8 weeks	Placebo	Consumed High molecular weight polyethylene glycol (HWPEG) 5 g twice/day	Significant difference (*p =* 0.05)	-Mean differences of FBG, HbA1c, cholesterol, and TG were significantly decreased in PSP group (*p* < 0.05). -Post-intervention values of FBG and HbA1c were significantly lower in PSP group (*p =* 0.02 and 0.01, respectively). -No significant differences in TC and TG (*p =* 0.51 and 0.26, respectively).	NA	C
28	60/48	48 T2D	30–75	Clinical/Okra powder	Consumedokra powder 10 g blended in 150 g yogurt along with dinner and lunch/8 weeks	Placebo	Consumed conventional yogurt alone, along with dinner and lunch	No significant difference	FBG (*p =* 0.02), HOMA-IR (*p =* 0.01), QUICKI (*p =* 0.004), TG (*p =* 0.001), TC (*p =* 0.004), LDL cholesterol (*p =* 0.02), and the ratio of LDL cholesterol to HDL cholesterol (*p* = 0.01) all demonstrated statistically significant reductions.	NA	C
29	70/60	60T2D	51	Clinical/saffron supplement	Consumed saffron supplement 100 mg/day /8 weeks	Placebo	Consumed starch 100 mg/day	No significant difference	-Significant reductions in TG levels by 22.72% and LDL cholesterol by 13.17% (*p =* 0.05). -Decreasing of nitric oxide and malondialdehyde by 26.29 and 16.35%.	Clinical	C
30	116/110	110 T2D	49–51	Clinical/ Ziziphus jujube Fruit (ZJF)	Consumed ZJF 300 mL/day before main meals balanced diet with 55% carbohydrate, 15% energy protein 30% energy fat	Placebo	Balanced diet (500 kcal/day deficit from estimated energy requirements) with (55% carbohydrate, 15% energy protein 30% energy fat)	Significant improvement (*p =* 0.03)	-Statistically significant decrease in TC, TG, and LDL-C levels. -The ratios of LDL-C to HDL-C and TC to HDL-C were significantly reduced. -Statistically significant based on the following p-values: TC (*p =* 0.02), TG (*p =* 0.05), LDL-C (*p =* 0.01), LDL-C/HDL-C ratio (*p =* 0.01), and TC/HDL-C ratio (*p* = 0.02).	NA	C
31	34/30	30T2D	49–57	Clinical/Black tea	Consumed three cups (three tea bags in 600 mL)/day/3 weeks	Intervention	Consumed one cup (one teabag in 200 mL)/day	Significantly lowered HbA1c levels in higher intake group (*p* < 0.05)	-Higher levels of regulatory T cells (Tregs), including CD3 + CD4 + CD25 + FOXP3 and CD3 + CD4 + IL-10 + cells.	NA	C
32	60/54	54T2D	53–55	Clinical/ caper fruit extract	Consumed 400 mg caper fruit extract (1200 mg hydro-alcoholic caper fruit extract = 5 g dry caper fruit) three times a day	Placebo	Placebo capsule three times/day	Significantly decreased HbA1c (*p* = 0.043)	-Significantly decreased in FBG level (*p* = 0.037) and serum TG level (*p* = 0.29) compared with control group.	NA	C
33	22	22 T2D	68–69	Clinical/ Beta glucan	Consumed functional bread, low in starch, rich in fibers (7 g/100 g) with a beta glucan/starch ratio of (7.6:100, g/g)	Clinical/ 24 weeks	Consumed regular white bread	Significant reduction (*p* = 0.027).	-Experimental group has better post-prandial plasma glucose (*p* = 0.011) greater TG concentrations (*p <* 0.015) when compared to the control group.	NA	C
34	40/32	32 T2D	60–61	Clinical/ Flaxseed oil	LA rich (~57.2 weight %) flaxseed oil (60 mg ALA/kg body weight/ day)	Clinical/ 12 weeks	Approximately 103 mg of safflower oil/kg body weight/day	NA	-Homeostatic high doses of flaxseed oil have no statisticallysignificant effect on HOMA-IR or HOMA-%β.	3 months	C
35	70/70	70 T2D	50	Clinical/ Soy nut diet	Consumed 60 g soy nut diet in two inter-meals of morning and afternoon	Placebo/ 8 weeks	The usual diet	NA	-Significantly decreased FBG (*p* = 0.03) TC (*p* < 0.01), LDL-c (*p* = 0.01), and E-Selectin (*p* < 0.01). -Increased capacity of serum total antioxidants (*p* < 0.01), brachial blood flow (*p* < 0.01).	NA	C
36	26/26	26 T2D	50–55	Clinical/ Aged garlic extract (AGE)	Consumed (AGE) (kyolic) 4 capsules/day (1200 mg) for 4 weeks then a 4-week washout period then placebo for 4 weeks	placebo 4 weeks, 4 weeks wash out, then 4 weeks	Daily consumption of Placebo for 4 weeks, nothing 4 weeks, then 4 weeks with placebo	No significant effect	No clinical benefit of adding AGE, in the short term.	NA	C
37	42/42	42 T2D and Hypertensive	64–66	Clinical/ Flavanol-rich cocoa	Consumed capsules with 2.5 g/day of a flavanol-rich cocoa (ACTICOA™ cocoa)	Placebo/ 12 weeks	Consumed cocoa-free capsules (4 capsules/ day)	No significant difference	-Does not appear to have any significant impact on blood pressure, blood sugar levels, or the way the body processes fats.	NA	C
38	49/45	45T2D	57–67	Clinical/ Resveratrol	Consumed resveratrol (99% pure trans-resveratrol; Mega Resveratrol, Southampton, UK) 400 mg capsules twice a day/8 weeks	Placebo/ 8 weeks	Placebo capsule (Completely inert micro cellulose)	Significantly reduced (*p* < 0.001)	Reduced FPG (*p* < 0.001), 2 h plasma glucose (*p* < 0.001).	NA	C
39	27/27	27 T2D	61–62	Clinical/Oat-enriched diet	-Managed diet and lifestyle only for two consecutive 8-week periods following with the oat-enriched diet containing 30% oats (selected commercially available oat-based products including cereals, oatcakes, bread, cereal bars) with lower saturated fat and refined sugar contents at each meal with an oat-based product, to include 60–100 g of oats per day	Managed diet and lifestyle only for two consecutive 8-week periods following withre-enforced standard dietary advice	Consumed 400 mg completely inert micro cellulose capsules, twice a day	NA	-An oat-enriched diet did not affect blood sugar control or insulin levels after a meal. -Diet did lead to a small decrease in TC levels (*p =* 0.019). -Levels of adiponectin, a hormone involved in regulating blood sugar and fat metabolism, decreased after a meal (*p* = 0.009).	NA	C
40	180/180	180 Impaired glucose tolerance	60–70	Clinical/Cereal fiber supplement from oat hulls	The 12-month PREDIAS intervention included 12 two-hour sessions: eight core lessons over eight weeks, and four booster sessions over the following ten months. Participants received an insoluble cereal fiber supplement from oat hulls; Vitacel OF 560-30 ; Rettenmaier &Söhne (70% cellulose, 25% hemicellulose, 3–5% lignin)	Placebo/ 2 years	A 12-month lifestyle intervention (PREDIAS) included eight core 2-h lessons over 8 weeks, followed by four booster sessions over the next 10 months, and then a 2-year placebo period	NA	Both groups saw lower 2-h oral glucose tolerance test (OGTT) levels after one year, but the difference between the groups was not significant.	NA	C
41	54/49	49 Obese T2D	49–62	Chicory oral	Chicory inulin enriched with oligofructose 10 g/day (Frutafit IQ, Sensus, Borchwef 3, 4704 RG Roosendaal, The Netherlands) for 8 weeks	Placebo	Maltodextrin 10 g/day (Jiujiang Hurirong Trade Co., Ltd., JiuJiang city, Jiangxi, China)	Intervention 7.74 ± 0.75 Control 8.43 ± 1.06, *p =* 0.001	SBP 004 vs. 0.048	NA	C

Legend for acronyms. HbA1c = glycated hemoglobin, the average level of blood glucose over the past 2–3 months; N/A = Not available data.

## Data Availability

The original contributions presented in the study are included in the article: and further inquiries can be directed to the corresponding authors.

## Supplementary Materials

The following supporting information can be downloaded at: https://www.mdpi.com/article/10.3390/foods14111919/s1, Full Search Terms.
